# Auditory Cortical Changes Precede Brainstem Changes During Rapid Implicit Learning: Evidence From Human EEG

**DOI:** 10.3389/fnins.2021.718230

**Published:** 2021-08-16

**Authors:** Erika Skoe, Jennifer Krizman, Emily R. Spitzer, Nina Kraus

**Affiliations:** ^1^Department of Speech, Language and Hearing Sciences, Connecticut Institute for Brain and Cognitive Sciences, University of Connecticut, Storrs, CT, United States; ^2^Auditory Neuroscience Laboratory, Department of Communication Sciences, Northwestern University, Evanston, IL, United States; ^3^Department of Otolaryngology, Head and Neck Surgery, New York University Grossman School of Medicine, New York, NY, United States; ^4^Department of Neurobiology and Physiology, Northwestern University, Evanston, IL, United States; ^5^Department of Otolaryngology, Northwestern University, Evanston, IL, United States; ^6^Institute for Neuroscience, Northwestern University, Evanston, IL, United States

**Keywords:** auditory system, corticofugal, online learning, frequency following response (FFR), statistical learning

## Abstract

The auditory system is sensitive to stimulus regularities such as frequently occurring sounds and sound combinations. Evidence of regularity detection can be seen in how neurons across the auditory network, from brainstem to cortex, respond to the statistical properties of the soundscape, and in the rapid learning of recurring patterns in their environment by children and adults. Although rapid auditory learning is presumed to involve functional changes to the auditory network, the chronology and directionality of changes are not well understood. To study the mechanisms by which this learning occurs, auditory brainstem and cortical activity was simultaneously recorded *via* electroencephalogram (EEG) while young adults listened to novel sound streams containing recurring patterns. Neurophysiological responses were compared between easier and harder learning conditions. Collectively, the behavioral and neurophysiological findings suggest that cortical and subcortical structures each provide distinct contributions to auditory pattern learning, but that cortical sensitivity to stimulus patterns likely precedes subcortical sensitivity.

## Introduction

Natural sound environments are rich with temporal and spectral patterns that repeat over different timescales. To extract these patterns, the brain must analyze the soundscape to learn about its statistical properties, including the probability that two sounds repeatedly co-occur. This analysis happens rapidly and often without conscious awareness. Evidence of rapid neural computations relating to predictive coding can be observed across the central auditory network, from brainstem to auditory cortex (Carbajal and Malmierca, 2018). Within the auditory system, brainstem and cortical structures also operate reciprocally through ascending and descending pathways (Winer, 2006). Through the descending corticofugal pathway, the auditory cortex can alter the input it receives, inducing short-term changes and long-term subcortical reorganization that either facilitate or attenuate subcortical processing of specific stimulus features (Suga et al., 2002). The corticofugal system appears to play an important role in auditory learning (de Boer and Thornton, 2008; Bajo et al., 2010). However, how learning ultimately emerges from these network processes is poorly understood, and questions remain about the degree to which brainstem and cortical structures independently, or dependently, contribute to different learning stages.

Pattern learning (“statistical learning”) is viewed as a general-purpose mechanism that underlies language and music learning (Saffran et al., 1997; Saffran, 2003; Misyak and Christiansen, 2012). Despite significant behavioral evidence of statistical learning, neurophysiological investigations of human auditory learning rarely examine this type of learning and when the neurophysiological correlates of short-term auditory learning have been investigated, they generally focus on cortical (Tremblay et al., 2001; Ben-David et al., 2011) or subcortical structures (Hornickel et al., 2012; Song et al., 2012) in isolation. Here we focus on both. To study the neural correlates of rapid pattern learning we coupled behavioral measures of learning with electroencephalogram (EEG) recordings, using an approach that allowed us to extract cortical and subcortical activity from the same EEG recording (Font-Alaminos et al., 2021). EEG was recorded while adult humans passively listened to continuous sequences comprised of eight musical tones (C4, D4, E4, F4, F#4, G4, G#4, and A4) ranging in fundamental frequency (F0) (262–440 Hz). Sequences were designed so that the transitional probability (TP) between tones (i.e., the probability that one tone followed another) was either randomized to create an “unpatterned” condition or fixed to create “patterned” conditions. Two patterned conditions were used in the experiment, with different participant groups receiving each. These patterned conditions were created by pairing the eight tones into four doublets and fixing the doublet TP at 100% (e.g., C4 always followed E4). For these two patterned conditions, the same eight tones were used but the doublet set did not intersect. The inter-stimulus interval was the same between the patterned and unpatterned conditions so that the doublets in the patterned conditions could only be detected by their TPs and not conspicuous breaks between doublets. Despite having the same short- and long-term TPs, one of the patterned sets was harder to learn (Skoe et al., 2015). By comparing these two patterned conditions of varying difficulty, we aimed to capture auditory system plasticity at different stages of the learning process, while preserving stimulus features like F0, TPs, and interstimulus interval.

In EEG recordings, activity from various neuronal sub-populations is pooled into a single waveform. By selective signal filtering, low-frequency cortical potentials (<30 Hz) like the P1-P2 response, can be separated from the higher-frequency phase-locked response characteristic of brainstem activity (Skoe and Kraus, 2010). Tones, such as those used here, elicit transient responses time-locked to the stimulus onset and tonic responses time-locked to the F0 (“frequency following response,” FFR). P1 and P2 are transient responses arising ∼100 and ∼200 ms (respectively) after the stimulus onset. They are generated in or near the primary auditory cortex, with the P2 generators extending to secondary auditory cortex and auditory association areas (Picton and Hillyard, 1974; Ponton et al., 2002; Martin et al., 2008). P1 and P2 are both sensitive to learning effects (Tremblay and Kraus, 2002; Ben-David et al., 2011), however, they have different maturational time courses (Ponton et al., 2002; Sussman et al., 2008), suggestive of unique neural processes. While P1 and P2 are distinguishable experimentally and developmentally, their unique functional significance is poorly understood, in part because P1 is generally small in adults (at the long interstimulus intervals usually used for cortical AEPs, >0.5 s) and because P2 typically co-varies with N1 (Crowley and Colrain, 2004). Like onset responses, tonic responses like FFRs can be observed across the neuro-axis. For frequencies >200 Hz, cortical phase-locking is weak to non-existent and thus FFRs measured to the frequencies used here likely reflects predominantly brainstem sources (Coffey et al., 2019; White-Schwoch et al., 2021).

Our previous report on this dataset focused on the FFR (Skoe et al., 2015). We found that the FFR to the patterned condition was different (smaller) from the unpatterned (baseline) condition, but only for the easier and not the harder condition. We now follow up on this finding by extracting and analyzing the P1-P2 cortical responses, which were recorded simultaneously with the FFRs. The goal was to use the combination of cortical (P1-P2) and brainstem (FFR) responses to better understand possible top-down, cortically driven, effects on the FFR during a short-term learning paradigm. We used task difficulty as a window into the directionality, and possible chronology, over which learning takes place across the auditory network. For the easier and harder conditions, different amounts of learning took place over the same exposure, creating the experimental conditions for studying EEG from brainstem and cortex at different stages of learning: a more advanced stage for the easier condition and an earlier stage for the harder condition.

## Materials and Methods

Thirty-six young adults, ages 18–26, participated in the learning paradigm. Written informed consent was obtained from all, with experimental protocols approved by Northwestern University’s Institutional Review Board. Before testing, participants were pseudo-randomly grouped into two groups (*n* = 18/group). An additional 18 participants were tested on a control condition to confirm that the FFR, P1, and P2 components did not change upon repeated presentation of the unpatterned condition. The three groups were age and gender matched. Groups were also statistically matched with respect to pure tone hearing thresholds in the 250–8kHz range, auditory brainstem response Wave V latency, IQ, auditory working memory, total years of musical training, and performance on a musical skills test. The details of this can be found in our earlier report on this dataset, see Skoe et al. (2015).

**FIGURE 1:**
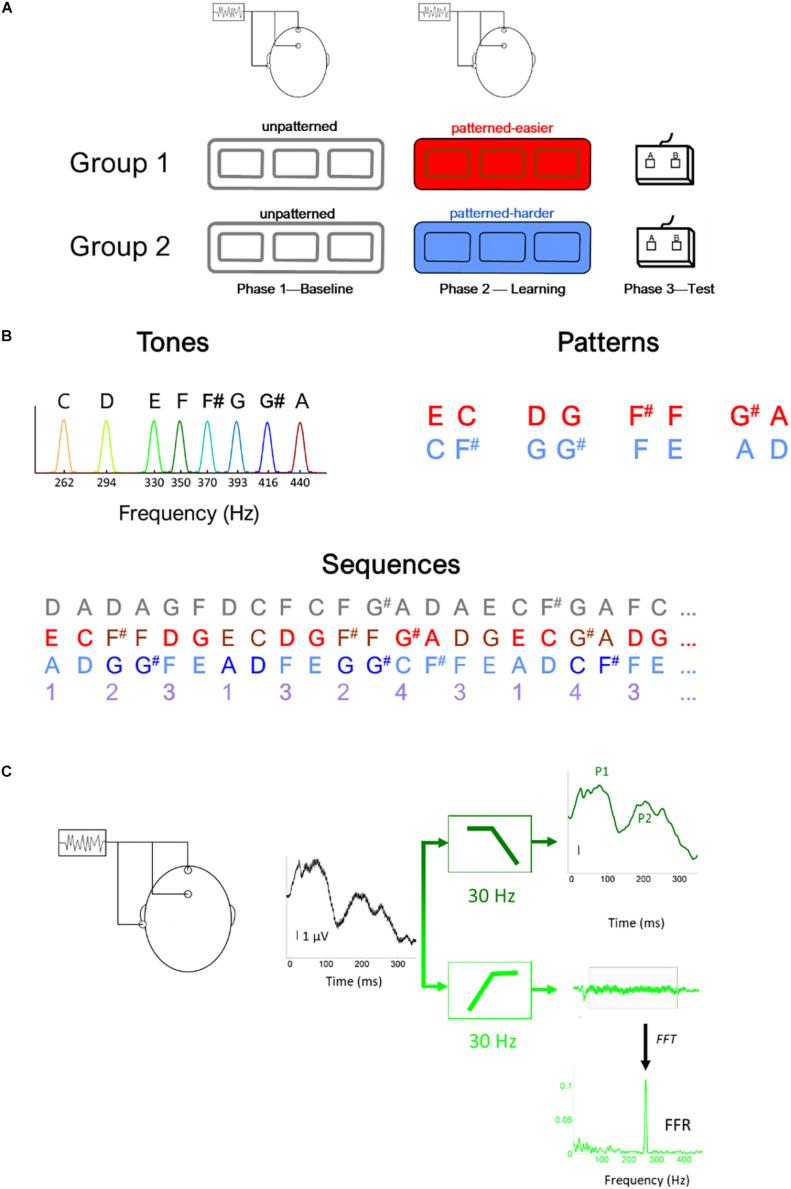
Illustration of **(A)** experimental design, **(B)** stimulus (i.e., tones) and sequence characteristics, **(C)** filtering procedure to derive the brainstem (light green) and cortical (dark green) potentials, illustrated using the response to the lowest tone (262 Hz) **(C)**. In panel **(B)**, the gray numbers illustrated the unpatterned sequence and the purple numbers illustrate the generating sequence used to construct both the easier (red) and harder (blue) sequences. Using this generating sequence, 1 was replaced with EC for the easier sequence and AD for the harder sequence and 2 was replaced with F#F in the easier sequence and GG# in the harder sequence, etc. Thus, in addition to being composed of tones with the same fundamental frequencies and the same interstimulus interval between tones, the generating structure of the two pattern sequences had the same transitional probability distribution.

The study included three phases (Figure 1A): Phase 1: Baseline EEG, Phase 2: Learning phase, and Phase 3: Testing phase. During Phase 1, EEG was recorded to the unpatterned condition to establish baseline levels of activity. During Phase 2, EEG was recorded while participants listened to one of two different patterned conditions, as part of an implicit learning paradigm. In Phase 3, participants were tested behaviorally on whether they could recognize the patterns heard during Phase 2 and their performance served as behavioral measure of implicit learning. We opted to fix the presentation order of the unpatterned and patterned conditions rather than use an interleaved or counterbalanced order because of concerns that an interleaved order would interfere with implicit learning, and concerns that the patterned condition could influence the unpatterned (baseline) condition if presented first (Weiss et al., 2009).

At the outset of the Learning Phase, participants were told to listen carefully to the sounds and that they would later be tested on how well they remembered the sounds. To facilitate alertness while minimizing muscle movement, participants watched nature photos. Because statistical learning can be interrupted by a concurrent attention-demanding task (Toro and Trobalon, 2005), participants did not perform a photo-related or secondary task.

All three conditions (unpatterned and two patterned) were formed from the same eight complex tones (333 ms each), played at the same rate (2.7 tones/s, ISI = 37 ms). The F0s of the eight tones were 262, 294, 330, 350, 370, 393, 416, and 440 Hz with each tone mapping to a specific musical note (C4, D4, E4, F4, F#4, G4, G#4, and A4, respectively). The tones were triangle waves containing odd harmonics of the F0, where each successive harmonic diminished in amplitude by 1/X (X = harmonic number). These triangle wave stimuli were chosen because their natural clarinet-like sound quality is more pleasant to listen to than pure tones and because their spectral profile produces robust FFRs, especially when a small number of trials are used (Jeng et al., 2011; Tichko and Skoe, 2017).

Across the three conditions, each of the eight tones was presented with the same overall probability but different TPs. For the unpatterned condition, the TP was pseudo-randomized so that each tone had a roughly equal probability of being followed by another but could not follow itself (1/7 or 14.3% TP). See Figure 1B for an illustration of the first ∼8 s of each sequence. For the patterned sequences, tones were presented in pairs, and each pair was drawn from a pre-arranged set of four options, without direct repetition. The tone pair set was unique for each patterned sequence: Easier [EC, F#F, DG, G#A] or Harder [AD, G#G, FE, CF#]. The TP for each tone pair was 100%. During the Baseline and Learning Phases, the sequences were presented as 5-min blocks, with short 1-min breaks between blocks. Within each block, each tone was presented 100 times, for a total of 300 presentations. The experimental design included three blocks with the initial intention of studying the time-course of plasticity. While pilot testing suggested that 100 trials were sufficient to elicit robust FFRs, this did not bear out in the full study sample, where we found that 300 stimulus presentations needed to be averaged for the FFRs to the highest stimulus frequencies to be above the noise floor for many of the participants in the sample.

During the Testing Phase, participants were given a two-alternative forced-choice test in which each tone pair from the patterned sequence was presented with a foil pair, two sounds that were heard but never sequentially during the Learning Phase (Saffran et al., 1999; Abla et al., 2008). Participants were instructed to select the more familiar-sounding pair. Each pair was tested against four foils creating 16 comparisons, with each comparison tested once. The pairs forming one sequence were inverted to create the foils for the other. For example, the tone pair EC in one sequence was inverted to create the foil CE for the other sequence. Scores were converted to percent correct, with 50% representing chance. Pilot testing showed that the sequence comprised of [EC, F#F, DG, G#A] produced higher test scores [independent *t* test: *t*(26) = 3.595, *p* = 0.001], motivating us to label this sequence as “easier” and the other as “harder.” This condition difference holds for the current dataset as well. In our previous publication of this dataset (Skoe et al., 2015), we reported that performance was at 61% correct for the easier condition compared to 53% for the harder condition. For the easier sequence, 15 of 18 (83%) participants performed above chance (i.e., 50%) compared to eight of 18 for the harder sequence. Within the easier sequence, F#F and G#A were more easily remembered than the other two tone pairs. While both sequences were novel and had similar TP distributions, the easier one was judged to be more musical by highly trained musicians tested during the pilot stage. This may account, at least in part, for why performance was different between the two sequences.

### EEG Protocol

Electroencephalogram was recorded with an analog-to-digital rate of 20 kHz (SynAmps 2 amplifier, Neuroscan Acquire, Compumedics, Inc.). Three Ag-AgCl electrodes were placed on the scalp (non-inverting electrode at Cz, inverting electrode at A2, ground at FPz), with contact impedance <5 kilo ohms. Recordings were made in continuous mode with an online filter of 0.5–3,000 Hz and then were processed offline in Neuroscan Edit. An offline low-pass filter (<30 Hz, 12 dB/octave) isolated the cortical onset components of the recording. To extract the FFR, a 30–2,000 Hz (12 dB/octave) offline filter was applied (Figure 1C). After filtering, recordings were epoched with a window of −10 to 350 ms surrounding the onset presentation of each tone, and then baseline corrected to the mean voltage of the noise floor (−10 to 0 ms) before applying threshold-based artifact rejection criterion (FFR ± 35 μV, cortical response ± 100 μV). For each of the eight tones, 300 artifact-free trials were averaged for each participant (100/block) for the FFR and P1-P2 recordings.

The sustained component of the FFR (55–278 ms) was converted to the frequency domain using a fast Fourier transform. The FFR amplitude at the F0 of each tone was extracted following previously described procedures (Skoe et al., 2015). Our previous report showed that while some of the tone pairs were easier to remember than others in the easier condition, this was not reflected in the FFR of individual tones. Instead, the FFR effect emerged as a global reduction in amplitude across frequency. This prompted our focus on global and not tone-specific effects here. Because of this focus, the FFR-F0 amplitude was averaged across the eight tones to obtain a single value representing the response to the unpatterned and patterned sequences for each participant. Similarly, for the P1 and P2 analysis, P1 and P2 amplitudes were derived from the responses to the individual tones and then averaged to create a single value for each condition. For P1, the average amplitude was calculated over 60–85 ms and for P2 the average amplitude was calculated over 160–220 ms. At the relatively fast rate of presentation used here (2.71 tones/s), P1 and P2 are the most prominent waveform components; N1, which is generally quite large in adults at slow presentation rates, is attenuated at faster rates (Sussman et al., 2008), and so was not analyzed here.

### Statistical Analysis

Linear mixed-effects models were used to test for the effect of group and condition (patterned vs. unpatterned) (FFR amplitude, P1 amplitude, P2 amplitude), using subject ID as the random intercept. Statistical analyses were performed in MATLAB version R2019b using the function *fitlme* with the default covariance matrix structure (full covariance) and fit statistic method (maximum likelihood).

## Results

**FIGURE 2:**
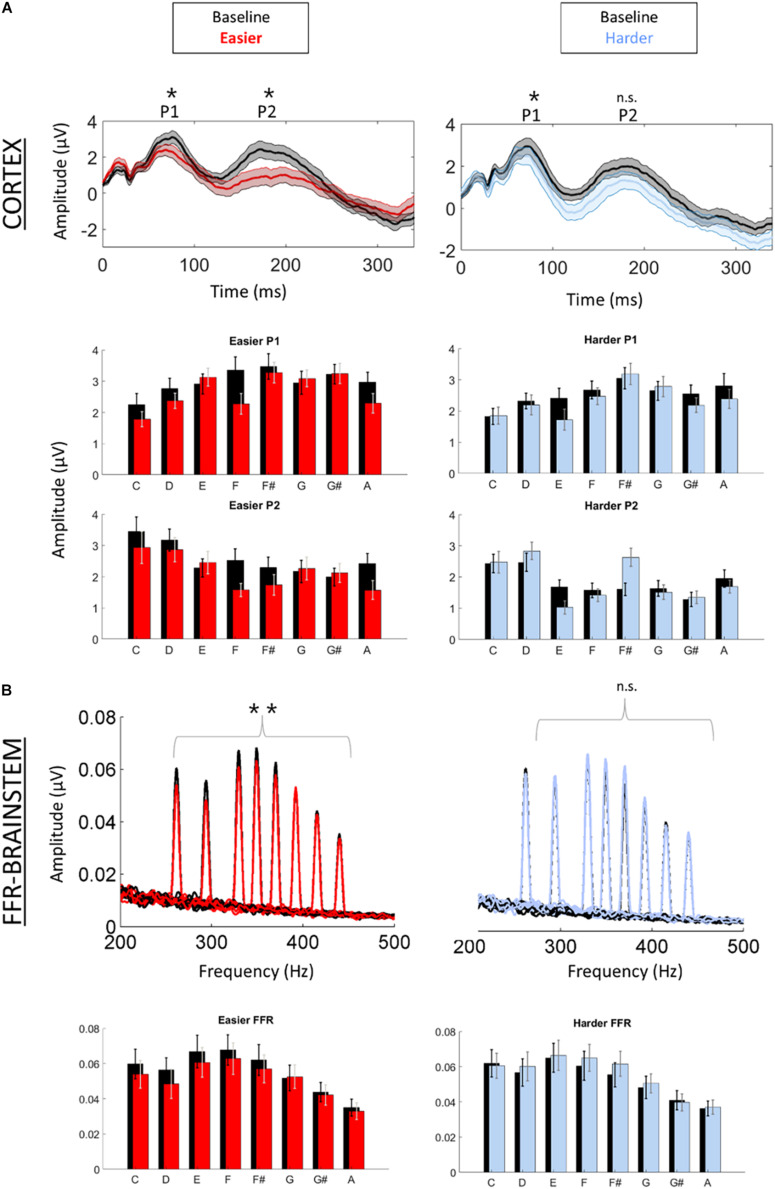
Cortical (A) and brainstem (B) responses were recorded simultaneously while participants listened to a baseline (unpatterned) condition (black) followed by a patterned condition (red = easier, blue = harder). **(A)** Time-domain cortical evoked potentials are plotted for the baseline and patterned conditions (averaged across all tones). Shading represents + /1 standard error of the mean (SEM) amplitude. Peaks P1 and P2 are labeled. Bar graphs of the mean amplitude for each tone for P1 and P2 are plotted below the time domain waveforms. Error bars represent ± 1 SEM. **(B)** Brainstem frequency-following responses are plotted for each of the eight tones across both conditions. Bar graphs of the mean amplitude of the FFR for each tone are plotted below waveforms. Error bars represent ± 1 SEM. *p < 0.05; **p < 0.01.

**FIGURE 3:**
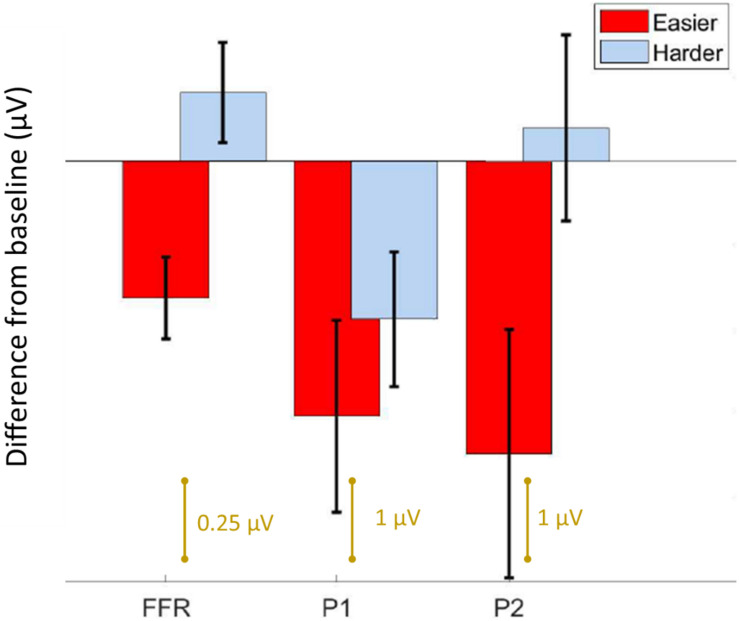
Summary of results. Bar graph showing the average difference from baseline ± 1 standard error of the mean for the FFR, P1, and P2 components. For the easier condition, both the brainstem and cortical components are reduced from baseline, whereas for the harder condition, only P1 is different. Note the FFR is smaller in amplitude than the cortical components, and is plotted on a different scale.

We first confirmed that repeating the unpatterned condition twice did not change the FFR, P1, or P2 components for the control group [*FFR t*(34) = 1.24, *p* = 0.23; *P1 t*(34) = 0.14, *p* = 0.89; *P2 t*(34) = −1.82, *p* = 0.11; example model formula = “*FFR*∼*1* + *condition* + (*1| ID*)”]. Next, we confirmed the response to the unpatterned condition was not statistically different between the experimental groups who received the easier vs. harder patterned conditions [*FFR t*(34) = 0.40, *p* = 0.69; *P1 t*(34) = −0.61, *p* = 0.54; *P2 t*(34) = −1.17, *p* = 0.25; example model formula = “*FFR*∼*1* + *Group* + (*1| ID*)”]. From there, we compared the two patterned conditions by testing whether the groups differed with respect to how much the amplitude changed between the unpatterned and patterned conditions [example model formula = “*FFR-change*∼*1* + *Group* + (*1| ID*)”]. This analysis revealed that the easy and harder conditions differed for the FFR-change and P2-change measures, but not the P1-change [FFR *t*(34) = −3.27, *p* < 0.01; P1 *t*(34) = −0.84, *p* = 0.40; P2 *t*(34) = −2.16, *p* = 0.04] (Figures 2, 3). Moreover, within-group comparisons between the patterned and unpatterned conditions showed that all three evoked responses were statistically smaller than baseline for the easier condition [FFR *t*(34) = 3.45, *p* < 0.01; P1 *t*(34) = 2.75, *p* < 0.01; P2 *t*(34) = 2.44, *p* = 0.02]. Figure 3 shows the mean change across the eight tones for the three evoked responses for the easier and harder conditions. To illustrate that the reduction for the easier condition is global and no systematic tone-specific effects are apparent, the means are also graphed for the patterned and unpatterned conditions for each tone in Figure 2.

For the harder condition, *only* P1 was statistically different (smaller) from baseline [*FFR t*(34) = −1.41, *p* = 0.17; *P1 t*(34) = 2.42, *p* = 0.02; *P2 t*(34) = −0.36, *p* = 0.72] (Figure 2). This P1-effect for the harder condition was driven by the eight participants who performed above chance on the harder condition. For the 10 “non-learners” where performance was below chance, P1 in the patterned condition did not differ from P1 in the baseline condition [*t*(18) = −0.14, *p* = 0.338] but for the eight “learners” it did [*t*(14) = 3.74, *p* < 0.01]. Outside of showing different patterns for P1, the learners and non-learners did not differ with respect to FFR or P2 (both *p* > 0.05). Despite this group difference, the correlation between the P1-change and performance was not significant (*r* = 0.12, *p* = 0.65).

## Discussion

Using EEG, we measured auditory brainstem and cortical activity during an implicit learning paradigm. Similar to previous studies of rapid learning (Abla et al., 2008; Alain et al., 2010; Ben-David et al., 2011), including a recent FFR study (Elmer et al., 2017), learning was associated with reduced neural activity. Although learning-related reduction occurred to some degree for both the easier and harder conditions, the two stimulus conditions showed different learning-related effects with respect to the brainstem and cortical components. In the easier condition, the FFR as well as the P1 and P2 components, differed from baseline, suggesting learning-related changes to both brainstem and cortex. The magnitude of the effect is visually similar for the two cortical components in the easier condition. Yet for the harder condition, only P1 and not the FFR or P2, differed from baseline, and this effect was driven by the learners in the sample. Thus, while P1 emerged as a potential marker of learning in both conditions, the FFR and P2 emerged only in the easier condition where learning was robust across participants. Our findings converge with other work to suggest that predictive coding occurs both cortically and subcortically but that it varies in its representation across brainstem and different cortical regions (Nieto-Diego and Malmierca, 2016; Font-Alaminos et al., 2021), and that it reflects long-term experience with sound that involves complex computations that go beyond low-level stimulus characteristics like inter-tone TPs (Kraus, 2021). Our constellation of findings also paints a complex picture of the possible timeline and top-down directionality of auditory network changes during rapid learning and they support the idea that learning emerge from multi-level representations of stimulus coding (Carbajal and Malmierca, 2018).

Cortical and brainstem potentials are both known to be sensitive to top-down effects, such as visual processing load (Xie et al., 2018), however, the involvement of the corticofugal pathway in these top-down effects and the learning-related processes studied here, remains inferential due to the lack of anatomic precision of the EEG signal. Techniques with greater anatomic and temporal precision do exist for studying human medial olivocochlear efferents, the lowest branch of auditory efferents. Human studies of medial olivocochlear function suggest that learning is dependent on efferent activity (de Boer and Thornton, 2008) and that the time scale of top-down modulation is fast (milliseconds to seconds) (Zhao and Dhar, 2011). Collectively, this supports the idea that top-down processes could potentially guide auditory learning over the brief timeframe of our rapid implicit learning paradigm (15-min of total exposure to the stimulus).

The important role of top-down processes in learning has been formalized in several popular models of learning and perception. The Reverse Hierarchy Theory of perceptual learning (Nahum et al., 2008; Ahissar et al., 2009), for example, proposes that perception is controlled by top-down, sequential processes whereby the perceiver first detects the gestalt structure and then only later, at more expert stages of learning, is aware of the compositional elements of the structure. The Reverse Hierarchy Theory further postulates that lower-level sensory changes arise only at more advanced learning stages and that through a “backward cascade” fine-grained sensory detail can be retrieved from lower neuroanatomical structures. This conceptualization of auditory learning as a backward propagation from higher to lower structures throughout learning is consistent with a recent study showing that sensory changes, measured *via* the FFR, did not emerge until learners were overtrained on the task (Reetzke et al., 2018). It is also consistent with evidence from motor learning, where early learning is mediated first cortically and later subcortically (Penhune and Doyon, 2002). If our results are interpreted under the Reverse Hierarchy Theory framework, P1 could be viewed as the antecedent in a chain of learning-related events, with changes to the FFR and P2 reflecting a later stage(s) of learning. If so, longer exposure to the harder condition might allow learning to progress to a later stage, with brainstem (FFR) changes eventually emerging.

The top-down sequential view of learning that we adopt is also in alignment with the Propagation Hypothesis, which posits that the memory trace for a sound is propagated to earlier processing stages each time the stimulus is presented (Baldeweg, 2006). This sequential framing of physiological changes also echoes results from the animal literature where subcortical and cortical experience-dependent plasticity occurred on different timescales (Lu et al., 2014), with cortical changes being antecedent to subcortical changes. While speculative, our findings could indicate that subcortical changes are subordinate to cortical changes and arise through top-down, cortically guided predictive coding processes during auditory learning. However, we offer this conclusion with some caution given that the timeline of changes was tested only indirectly through a stimulus comparison and not through a longitudinal design. We also note that the need to average across trials hindered our ability to directly study the timeline of changes within the existing dataset. It is also important to acknowledge that predictive coding in the brainstem diminishes but does not vanish when the cortex is de-activated (Anderson and Malmierca, 2013), which conflicts with a fully top-down account of auditory learning.

An alternative explanation for our findings is that cortical neurons are inherently more plastic and more sensitive to stimulus regularities, and therefore change before the brainstem, without brainstem changes necessarily being cortically guided. A recent study, however, complicates the conclusion that brainstem is necessarily slower to change than cortex (Elmer et al., 2017). In that study, participants underwent 1-h of phonetic discrimination training, with FFRs measured before and after training. Discrimination improved with training relative to a passive listening group, with greater improvement correlating with larger FFR suppression. However, in contrast to the FFR, no changes were observed to the mismatch negativity response (MMN), a cortically-generated response linked to stimulus specific adaptation in response to repeated stimulation (Nieto-Diego and Malmierca, 2016). Unlike our study which recorded FFRs and cortical potentials simultaneously during the learning phase, EEG wasn’t recorded during training, and the FFR and MMN were instead recorded sequentially using different stimulus paradigms. For the MMN, this involved an oddball paradigm using the end point stimuli of a phonetic continuum that produced robust MMNs even before training, suggesting possible ceiling effects for training on the MMN. The stimulus continuum was drawn from the participant’s native language (German), so previous familiarity with the stimuli could have influenced the physiologic results. By contrast the tonal sequences in our experiment were entirely novel. However, one of them sounded more musical, likely because the four tone pairs created musical motifs (combinations of sounds) that are common in Western music. This increased musicality, we speculate, may have facilitated predictive coding and sped up the learning process, allowing for more learning to take place within the same period (∼15 min) (Skoe et al., 2015; Carbajal and Malmierca, 2018). Despite these differences and limitations, the Elmer et al. (2017) study converges with our work to suggest that brainstem and cortex are differentially sensitive to short-term physiological changes related to auditory learning.

Our results provide the foundation for futures studies into the time course and directionality of changes within the auditory neural network during implicit learning. Although this line of research is admittedly still in elementary stages, and results should be confirmed in longitudinal designs, the preliminary data we present here reinforce that no single brain region provides a comprehensive chronicle of what is involved in auditory learning, that stimulus statistics are not redundantly represented across the auditory system, and that auditory learning proceeds in stages, with subcortical changes emerging later.

## Data Availability Statement

The raw data supporting the conclusions of this article will be made available by the authors, without undue reservation.

## Ethics Statement

The studies involving human participants were reviewed and approved by Northwestern University Institutional Board. The patients/participants provided their written informed consent to participate in this study.

## Author Contributions

ESk designed the study, with input from all authors, and wrote the manuscript, with input and feedback from all authors. ESk and ESp collected the data. All authors contributed to the article and approved the submitted version.

## Conflict of Interest

The authors declare that the research was conducted in the absence of any commercial or financial relationships that could be construed as a potential conflict of interest.

## Publisher’s Note

All claims expressed in this article are solely those of the authors and do not necessarily represent those of their affiliated organizations, or those of the publisher, the editors and the reviewers. Any product that may be evaluated in this article, or claim that may be made by its manufacturer, is not guaranteed or endorsed by the publisher.
